# How a State Measures Up: Ambulatory Care Pharmacists’ Perception of Practice Management Systems for Comprehensive Medication Management in Utah

**DOI:** 10.3390/pharmacy8030136

**Published:** 2020-08-01

**Authors:** Kyle Turner, Alan Abbinanti, Bradly Winter, Benjamin Berrett, Jeff Olson, Nicholas Cox

**Affiliations:** 1 Department of Pharmacotherapy, College of Pharmacy, University of Utah, Salt Lake City, UT 84112, USA; alan.abbinanti@pharm.utah.edu (A.A.); nicholas.cox@pharm.utah.edu (N.C.); 2 University of Utah Health, Salt Lake City, UT 84132, USA; golden.berrett@hsc.utah.edu; 3 Intermountain Pharmacy Services, Intermountain Health Care, Salt Lake City, UT 84102, USA; bradly.winter@imail.org (B.W.); jeff.olson@imail.org (J.O.)

**Keywords:** comprehensive medications management, practice management, ambulatory care, primary care, clinical pharmacy

## Abstract

Comprehensive medication management (CMM) is a patient-centered standard of care that ensures a patient’s medications are optimized. The CMM Practice Management Assessment Tool (PMAT) is a tool to assess areas of CMM practice management. The purpose of this project was to assess the state of CMM practice management based on clinical pharmacist perception for two health systems in the state of Utah, and to identify areas of excellence and/or improvement utilizing a novel method for PMAT analysis. The PMAT was distributed to all primary care-focused ambulatory care pharmacists employed by University of Utah Health (U of U Health) and Intermountain Healthcare (Intermountain). Ordinal responses were assigned to three possible categories of CMM support (High, Indifferent, and Low). Ten surveys were completed from U of U Health, and nine were completed from Intermountain. Thirty-two of the 86 survey questions resulted in a high level of support, and 25 questions resulted in a low level of support from the majority of respondents. Statistically significant differences between the institutions were found for 18 questions. The utilization of the PMAT within two Utah health systems highlighted areas of excellence and improvement and demonstrates a unique method for analysis of PMAT results.

## 1. Introduction

Comprehensive Medication Manage (CMM) is a patient-centered care process that ensures each patient’s medications are individually assessed to determine that each medication is indicated for a particular condition, is effective for the medical condition and achieving defined goals, is safe given the comorbidities and other medications being taken, and that the patient is able to take the medication as intended and adhere to the prescribed regimen [1,2]. CMM consists of three core elements: philosophy of practice, patient care process, and practice management system [1,2]. CMM has been shown to significantly improve clinical, financial, and humanistic outcomes when implemented [3,4,5,6,7,8]. Due to CMM’s beneficial impact on key outcomes, two institutions in the state of Utah—University of Utah Health (U of U Health) [9] and Intermountain Healthcare (Intermountain)—sought to implement CMM within primary care clinics at each health system. Recognizing that a functional practice management system was essential to effective implementation of CMM [10], the two health systems partnered to assess and compare results for insight into improvement strategies.

To aid the implementation of CMM, the Utah Alliance of Ambulatory Pharmacists (UAAP) was created as a practice advancement and advocacy learning collaborative to represent the voice of pharmacists in ambulatory care across the state. Representatives from U of U Health and Intermountain were founding members of UAAP. Utilizing the knowledge and relationships in UAAP, the two institutions partnered to improve their practice management systems in hopes of learning from one another and disseminating that learning to other members of UAAP. Inherent in this collaborative effort was the ultimate goal of practice management improvement within each organization, to gain the resources and essential elements of CMM needed to advance practice within the state.

Efforts to implement CMM through collaboration in UAAP centered on the work of the CMM in Primary Care Research Team [11,12], with the selection of the Practice Management Assessment Tool (PMAT) [12] as the instrument to determine the current state of practice management systems at the two institutions. The PMAT is a relatively new tool developed and validated by the University of Minnesota’s Department of Pharmaceutical Care and Health Systems to assess the level of support for CMM within an organization [12]. Due to its recent development, the use of the PMAT is limited and few articles have been published on its application in a novel environment. The purpose of this project was to assess the state of CMM practice management based on clinical pharmacist perception for two health systems in the state of Utah and to identify areas of excellence and/or improvement utilizing a novel method for PMAT analysis.

## 2. Methods

The PMAT consists of 86 questions regarding CMM support in various pharmacy practice areas, including 5 essential domains: Organizational Support, Care Team Engagement, Care Delivery Processes, CMM Program Evaluation, and Ensuring Quality Care. The first 5 questions use a Likert scale (from 1 to 10) to assess the 5 domains of CMM practice management’s performance and feasibility. The remaining 81 questions used a mix of categorical rankings to further assess the 5 domains, as well as essential components of CMM practice management. For example, a question asked, “Which of the following statements is most true for your practice site regarding availability of patient care space?”. Possible responses were “There is not a designated space and it is difficult to find space”, “There is not a designated space but it is not difficult to find space”, “There is a designated space”, “There are two or more designated spaces”. The PMAT was distributed to all primary care pharmacists within the two institutions to assess each individual practice. If multiple pharmacists or a pharmacy technician practiced within the site, one version of the PMAT was completed after input and collaboration from all involved.

Comparisons between questions or collaborating institutions could not be made using the native PMAT due to differing scales used for multiple questions. To overcome this issue, we created a uniform ordinal answer scale for all questions thus allowing for comparisons and determination of statistical differences. Each question’s possible responses were converted into an ordinal response of three possible groups: High, Indifferent, and Low support. Group categorization decisions were reviewed by a committee of 5 independent non-affiliated instigators who determined what possible responses were considered High, Indifferent, and Low for each individual question and its respective answer choices (See Supplementary Materials). For questions that only consisted of two possible answer choices, the Indifferent support category was excluded as a possible categorization. For questions that consisted of a “select all that apply” answer set, a range of selected choices was designated for each category (ex. High = 8–12, Indifferent = 4–7, Low = 0–3). For instance, in the above example question and answer set, responses indicating two or more spaces were considered High, having a dedicated space was indifferent and the two options stating there was not a dedicated space were considered Low, regardless if it was difficult to find spare or not. Each question was reviewed individually to create a unique categorization of its answers for High, Indifferent, and Low.

Surveys were distributed and collected via a paper copy in mandatory monthly meetings for clinical pharmacists at U of U Health, and via a Qualtrics survey at Intermountain. After surveys were completed, results were manually converted to the designated support categories. Comparisons between institutions were made using a Fischer exact test, due to a small sample size, and by comparing the number of High Support responses to non-High Support responses (i.e., Indifferent Support + Low Support)., and Low Support responses to non-Low Support responses (i.e., High Support + Indifferent Support). This was done to better identify differences in High and Low support by combining the Indifferent category with the opposite category being evaluated, and highlight areas of strength and opportunity between the institutions.

## 3. Results

All primary care pharmacy teams were given the PMAT and a 100% response rate was achieved, with a total of ten surveys completed from U of U Health and nine from Intermountain. Of the first five general questions regarding the five domains of CMM practice management, only one question demonstrated High Support responses from the majority of respondents (50% or more), specifically the feasibility of ensuring quality care. None of the first five general questions demonstrated Low Support from the majority of respondents (50% or more) or showed a statistically significant difference between the two institutions. The results are summarized in Table 1.

Thirty-two of the 86 survey questions resulted in a High level of support from the majority of respondents (50% or more) from both institutions (see Table 1). Key questions that demonstrated High levels of support include questions about adequate space for CMM activities, support from referrals, point of care testing, availability of equipment, support personnel, and scheduling and documentation. Twenty-five questions resulted in a Low level of support from the majority of respondents (50% or more) from both institutions (see Table 2). Key questions that demonstrated Low levels of support include questions about executive leadership support, collaborative care visits, image ordering, rooming and intake of patients, dedicated support personnel, and patient satisfaction feedback.

In comparing the two institutions, U of U Health showed statistically higher support than Intermountain in eight questions (*p* ≤ 0.0325), while Intermountain showed statistically higher CMM support in one question (*p* = 0.0055) (see Table 3). U of U Health showed higher support in areas of scheduling, point of care testing, support staff, and referrals, while Intermountain showed higher support for double documentation of visits. When evaluating Low support areas, U of U Health showed lower support in one question (*p* = 0.0055) regarding double documentation, while Intermountain showed lower support in eight questions (*p* ≤ 0.0325) about provider/pharmacist team satisfaction, data collection, and overall consistency (See Table 4).

## 4. Discussion

To our knowledge, this is the first paper published documenting the administration and analysis of the PMAT within health system primary care practices with novel CMM implementation and can be used to demonstrate its utility in assessing, planning for, and obtaining essential CMM resources. The initial utilization of the PMAT, and subsequent methodology for analyzing the results, uncovered several areas of excellence and improvement for each institution.

Areas of excellence include adequate space for CMM activities, referrals, point of care testing, equipment availability, support personnel, scheduling, and documentation. Areas where improvement is needed included rooming of patients, collaborative visits, dedicated support personnel, and a greater emphasis on patient feedback. The results will serve as a benchmark for both institutions and inform collaboration to advance practice. We anticipate that these results will be compared against future assessments utilizing the PMAT to determine progress in addressing identified areas for improvement. As a statewide ambulatory care collaborative, UAAP will use the results of this analysis to determine where state-level practice advancements are needed and lobby both institutional and government stakeholders for adequate resources.

Beyond collaboration, the utilization of the PMAT has created organizational and administrative changes within these institutions. For example, U of U Health has built the leadership structure for primary care services based on the five categories of CMM practice management and has identified stewards for the advancement of these areas. Moreover, Intermountain has begun pilot programs aimed at improving pharmacist satisfaction and modified certain data collection strategies.

The strengths of this paper include publication on the administration and analysis of the PMAT and offers a unique methodology for analyzing answers and comparing sites/institutions from the PMAT, a tool that was not initially designed for such investigation. This methodology can be used by other institutions that intend to utilize the PMAT to evaluate their CMM support.

Limitations include a small sample size making it difficult to meet power and detect differences between institutions for many questions. Another limitation of this paper is that the survey was utilized by only two health systems in one state. Further, variation in CMM practice history and institutional structure and priorities exist between the health systems that may account for differences in practice management systems.

This project highlights areas of excellence within Utah and multiple opportunities for improvement in practice management systems related to CMM implementation. Overall, health systems in Utah generally allow for High levels of practice through support for CMM. The results will be used to prioritize efforts to improve CMM implementation within healthcare institutions and within the state and will provide information about potential pitfalls in CMM practice management. It will also provide information on possible methods for future implementation and analysis of the PMAT to other healthcare systems nationally, allowing for greater development and advancement of CMM and enhanced collaboration and standardization of CMM practices.

## 5. Conclusions

The utilization of the PMAT within two Utah health systems highlighted areas of excellence and improvement within each institution and demonstrated a unique method for analysis of PMAT results. Results can be used by other systems and practices in their CMM implementation with regard to practice management. Overall, health systems in Utah generally allow for high levels of practice through support for CMM. The results will be used to prioritize efforts to improve CMM implementation through collaboration within both healthcare systems and will provide information to other healthcare systems nationally about potential pitfalls in CMM practice management.

## Figures and Tables

**Table 1:** Results of questions with High Support from the majority of respondents (50% or more).

PMAT Questions	Responses Categorized as High Support (%)
**Organizational Support**	
Which of the following statements is most true for your practice site regarding availability of patient care space?	58%
… availability of non-patient care space?	68%
… privacy of space?	74%
… size of space?	89%
… care space equipment?	89%
… clinical pharmacy leadership?	63%
**Care Team Engagement**	
… direct provider referrals?	63%
… placing new referrals to other care team members?	84%
… the ability to order labs?	95%
… the ability to order durable medical equipment? (e.g., blood pressure cuff)	68%
… point-of-care testing?	79%
**Care Delivery Processes**	
… patient identification for CMM services?	79%
… non-provider referrals?	74%
… generated quality care lists?	79%
… scheduling in EHR?	79%
… automatic appointment reminders?	68%
… scheduling assistance? (Clinic level scheduling)	74%
… scheduling assistance? (Reminder calls)	74%
… scheduling assistance? (Ensuring referrals get scheduled)	63%
… scheduling assistance? (Ensuring follow-up appointments get scheduled)	68%
… outreach? (Outbound calling)	53%
… system access to the documentation system?	95%
… double documentation?	53%
… completion of documentation?	58%
… documentation of Medication Therapy Problems (MTPs)?	63%
… documentation improvement initiatives?	74%
… the requirement of a physician’s co-signature?	100%
**CMM Program Evaluation**	
… the identification of Medication Therapy Problems (MTPs)?	63%
… aggregated-level data extraction?	58%
**Ensuring Consistent and Quality Care**	
On a scale of 0–10, with 10 being most feasible, how would you rate the feasibility of improving ensuring consistent and quality care in your CMM practice?	58%
… the process to ensure pharmacists are providing consistent and quality care?	53%
… the process to ensure notes have met documentation requirements?	74%

**Table 2:** Results of questions with Low Support from the majority of respondents (50% or more).

PMAT Questions	Responses Categorized as Low Support (%)
**Organizational Support**	
… clinic leadership?	53%
… executive leadership?	89%
**Care Team Engagement**	
… collaborative visits?	63%
… the presence of a champion?	58%
… orienting new care team members?	74%
… the ability to order imaging? (e.g., DXA * scan)	95%
… rooming patients?	84%
…the taking of vitals for patients during appointments?	79%
… billing and coding?	84%
… dedicated support personnel? (e.g., MA **, LPN ***)	58%
**Care Delivery Processes**	
… payer referrals?	84%
… appointment management? (check all that apply)	68%
… outreach? (Other mailings (e.g., brochure))	83%
… efficiency of inputting notes? (check all that apply)	65%
**CMM Program Evaluation**	
… the resolution of MTPs?	53%
… revenue generation?	53%
… estimated cost savings?	63%
… descriptive measures?	58%
… patient satisfaction?	100%
… patient-level data extraction?	53%
**Ensuring Consistent and Quality Care**	
… the training process for CMM philosophy of practice?	68%
… the training process for CMM patient care process?	58%
… the training process for CMM practice management?	58%
… for training?	63%
… the use of quality assurance processes for improvement?	53%

* Dual-energy X-ray absorptiometry; ** Medical Assistant; *** Licensed Practical Nurse.

**Table 3:** Statistically significant results of questions illustrating High Support.

PMAT Questions	Responses Considered High UofU * (%)	Responses Considered High IHC ** (%)	*p*-Value
… direct provider referrals?	90%	33%	0.0198
… point-of-care testing?	100%	56%	0.0325
… dedicated support personnel? (e.g., MA ***, LPN ****)	50%	0%	0.0325
… patient identification for CMM services?	100%	56%	0.0325
… scheduling in EHR?	100%	56%	0.0325
… scheduling assistance? (Centralized scheduling)	80%	11%	0.0055
… scheduling assistance? (Preparing patients for visit expectations)	80%	11%	0.0055
… scheduling assistance? (Ensuring referrals get scheduled)	100%	22%	0.0007
… double documentation?	20%	89%	0.0055
Totals	48%	31%	<0.00001

Note: Only questions with statistically significant differences were included. * University of Utah Health Care; ** Intermountain Health Care; *** Medical Assistant; **** Licensed Practical Nurse.

**Table 4:** Statistically significant results of questions illustrating Low Support.

PMAT Questions	Responses Considered Low UofU (%)	Responses Considered Low IHC (%)	*p-*Value
… scheduling assistance? (Centralized scheduling)	20%	78%	0.023
… double documentation?	80%	11%	0.0055
… clinical markers? (e.g., ACT * score, blood pressure, A1C **)	0%	56%	0.0108
… revenue generation?	10%	100%	0.0001
… estimated cost savings?	30%	100%	0.0031
… provider/team satisfaction?	0%	67%	0.0108
… the use of collected CMM data? (select all that apply)	0%	78%	0.0007
… the reporting of CMM data? (select all that apply)	0%	100%	0
… the process to ensure pharmacists are providing consistent and quality care?	0%	44%	0.0325
Totals	27%	46%	<0.00001

Note: Only questions with statistically significant differences were included; * Asthma Control Test; ** Percent Glycated Hemoglobin (Hemoglobin A1C).

## Supplementary Materials

The following are available online at https://www.mdpi.com/2226-4787/8/3/136/s1.
